# The Nature of Shell Shock

**Published:** 1919-01

**Authors:** 


**The Nature of Shell Shock.** By F. DILLON, Temp. Capt. R. A. M. C. *Bulletin of the Canadian Army Medical Corps*, Sept. 1918.

The author speaks of the diversity of opinion regarding the nature and origin of shell shock, which has been, since the outbreak of the war, attributed to neurological or mental disorders, or to a combination of both. He rejects the first and last suggestion and concludes that they are due to purely psychological troubles, explaining his views as follows:

The neurological hypothesis is untenable owing to: (1) the lack of direct evidence for the existence of an organic basis; (2) the sudden disappearance and mutability of symptoms; (3) the effect of psychological treatment in curing and ameliorating the symptoms; (4) the occurrence of precisely similar symptoms in men who had never been subjected to any exposure liable to cause organic damage.

The psychological explanation is found in the very nature of man. In every normal individual there is a latent tendency toward the arousal of certain emotions by certain stimuli. These emotions, which find their origin in man's instincts, — the combative, social, repulsive, fearful, etc., — tend, when aroused, to produce their characteristic effects. They are, on the other hand, counterbalanced by a more or less considerable amount of power of resistance. Under the long-continued strain of modern warfare, the reserves of mental energy gradually become exhausted. The balance is disturbed at a given moment, and an unexpected shock, i. e., a sudden inrush of the emotion-arousing stimulus, may cause a breakdown. The commonest form of war psychoneurosis is the " névrose d'effroi ", the symptoms of which are : headache, giddiness, mental confusion, visceral disorders, etc.; other forms show paralyses, contractures, etc. ; a third class consists of cases of complete amnesia and fugues ; a fourth group which may be called the " conversion " class, consists of cases in which the psychical trauma is converted into bodily symptoms: the fifth and last class is made up of the combined types, including, first, cases showing the symptoms of both neurological and psychoneurotic troubles; second, of war psychoneurosis which has developed in conjunction with pre-existing psychoneurosis.

*Prognosis and Treatment.* Shell shock, when treated with proper understanding, is, in the large majority of cases, not a serious condition. 70 o/o of the cases treated at advanced centers in France are made fit to return to duty.

The main principles of the treatment may be summarized as follows. The atmosphere of the wards should be one of understanding and of cure; an attitude of rational firmness must be maintained toward the patient from the outset; and the chief problem is to effect the rearrangement of the disorganized mental activities and to bring the conflicting forces in the mind into a state of equilibrium. This can be carried out by means of a system of mental analysis and individual re-education.

